# Revascularization of a Crushed Foot and Ankle Mortis

**DOI:** 10.7759/cureus.11561

**Published:** 2020-11-19

**Authors:** James El Haddi, Veronica Garbar, Lawrence Lottenberg, Robert Borrego, Mario Rueda

**Affiliations:** 1 Surgery, Florida Atlantic University Charles E. Schmidt College of Medicine, Boca Raton, USA; 2 Surgery, Florida Atlantic University/St. Mary’s Medical Center, West Palm Beach, USA; 3 Trauma, Florida Atlantic University/St. Mary’s Medical Center, West Palm Beach, USA

**Keywords:** trauma, extremity, vascular surgery, extremity trauma, crush injury, revascularization, amputation, limb salvage

## Abstract

Foot crush injury is a difficult problem both from the complexity of the injury pattern standpoint and also the significant clinical and socioeconomic burden that it represents to the patient. Scoring systems exist to predict limb salvage, but the accuracy and implementation of these are varied, and thus clinical judgment must always be employed when attempting limb salvage. This case report describes the first use of a reversed saphenous interposition graft repair of a transected dorsalis pedis in a patient after sustaining crush injuries to the distal lower extremity. The patient was able to undergo partial limb salvage with the use of revascularization and judicious fasciotomies.

## Introduction

Distal extremity crush trauma is often a challenge for management because orthopedic, soft tissue, vascular, and nerve injuries are frequently intermixed. Salvaging limbs following blunt distal extremity trauma is an extremely arduous process for the clinician, and the salvage rate is lower than penetrating injuries [1]. Though studies have shown that while early amputation may decrease hospital length of stay and re-admission [2], military data suggests that long-term psychological and functional outcomes may be similar regardless of salvage attempts [3]. In the civilian population, return-to-work times are of the utmost importance. These are dependent on multiple factors including the need for operative intervention, patient age, and location/extent of injuries [4]. Vascular injury can significantly affect rates of limb salvage and return-to-work times [5].

When evaluating in the trauma bay, multiple risk scores have been devised to predict which patients may have successful limb salvage [6-7]. Recent meta-analyses and retrospective studies have suggested that while popular scores such as the Mangled Extremity Severity Score (MESS) and Gustilo-Anderson classification have been widely accepted for risk stratification, they are often incorrectly employed and may have poor predictive value [7-8]. Unsuccessful limb salvage brings multiple interventions, hospital re-admissions, and delayed rehabilitation. Because of these risks, there is a need to predict which patients should even undergo initial attempts at limb salvage versus amputation. The rate of amputations for all causes (traumatic and nontraumatic) is as low as 43% [9] with quality of life and function dependent on the level of amputation [10]. Patients who suffer traumatic extremity injuries tend to be low-income, uninsured, and unskilled manual laborers, which further amplifies the socioeconomic burden [11]. In the following case, we describe the use of unique limb-salvage technique in a patient with a presenting MESS core of 6 and a Gustilo-Anderson score of 3b.

## Case presentation

**Figure 1:**
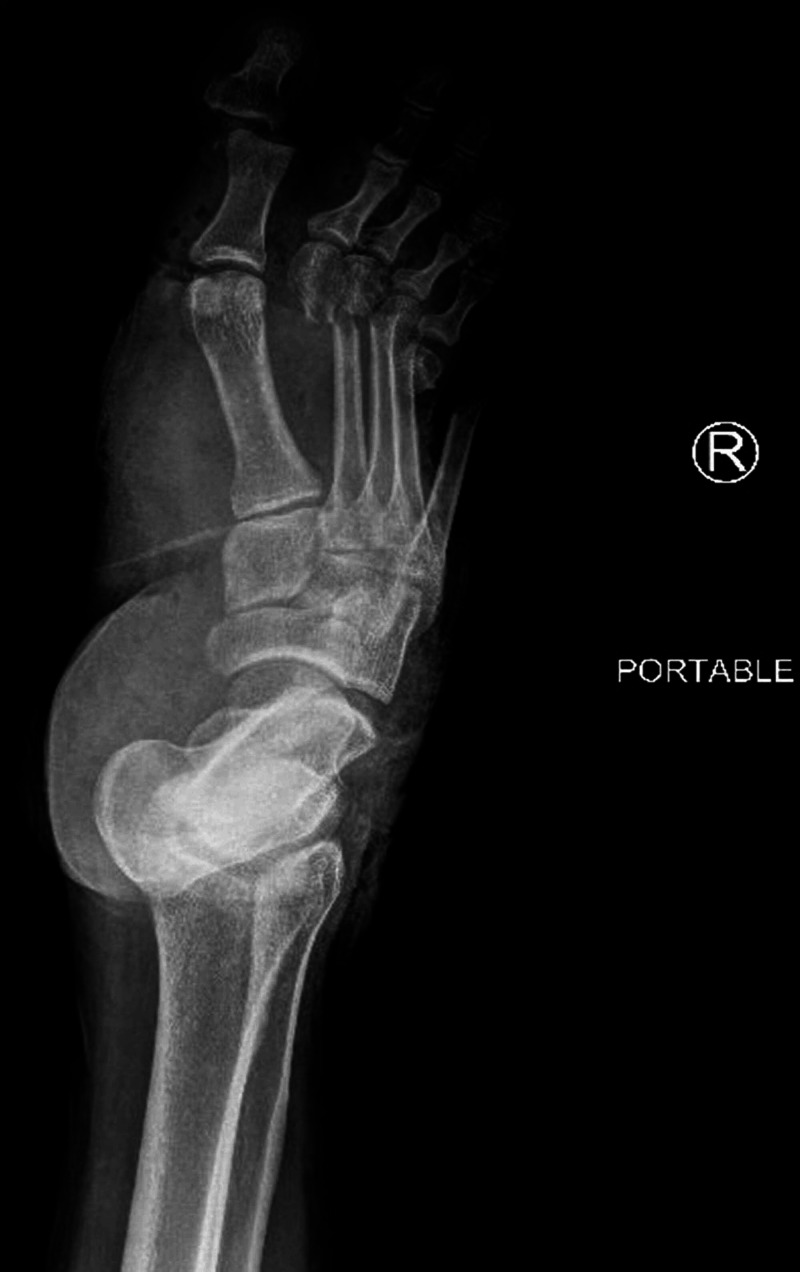
Plain film x-ray of the patient's crushed foot obtained in the trauma bay Plain film x-ray of the patient's right lower extremity. Note the displaced fractures of metatarsals 2-4, displaced right medial malleolar, and tibial metaphysis fractures.

**Figure 2:**
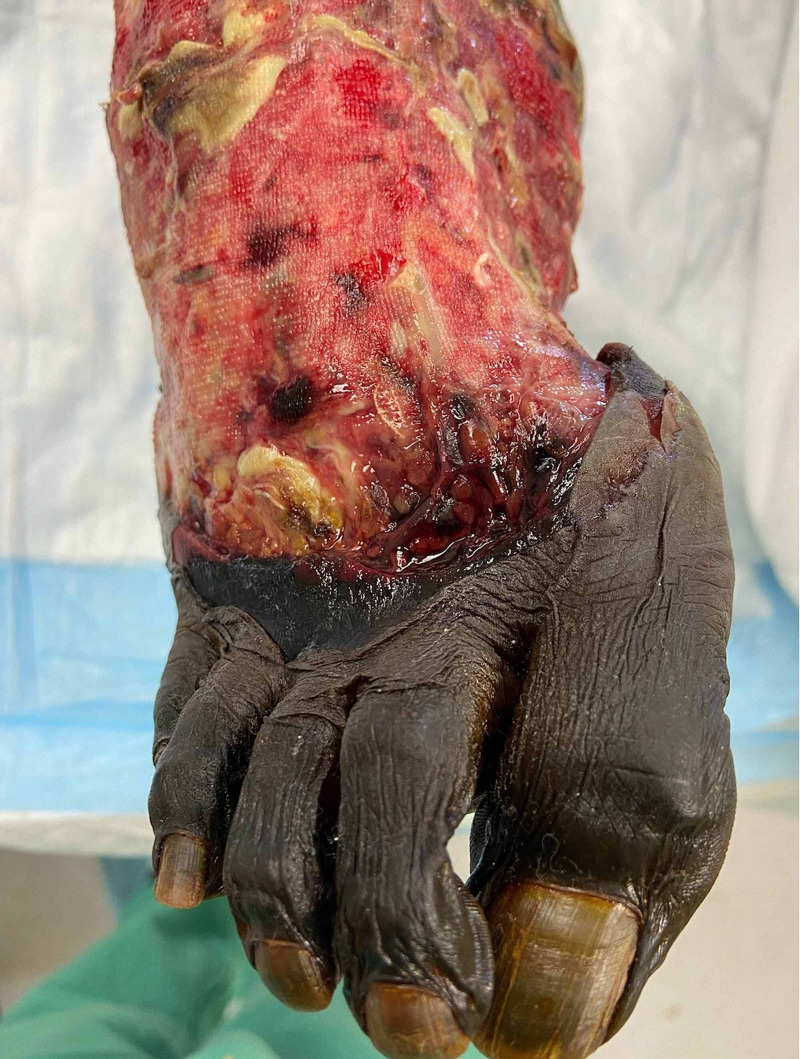
The patient's crushed right lower extremity prior to revascularization Here we see the patient's right lower extremity prior to revascularization. Significant nonviable tissue debridement and reduction of the open fractures had been completed by the orthopedic consultants.

The case in question involves a 44-year-old male who sustained a crush injury to both lower extremities after being run over by a large utility vehicle. In the trauma bay, he was noted to have a degloving injury of the distal right lower extremity in combination with an open fracture and non-pulsatile bleeding. Biphasic pedal signals were identified bilaterally during resuscitation. Plain films revealed open displaced fractures to right metatarsals 2-4 and displaced right medial malleolar and tibial metaphysis fractures with a spiral fracture of the proximal fibula as shown in Figure 1. His MESS score was 6, and Gustilo-Anderson score was 3b, which are not predictive of needing amputation but do predict an elevated risk of complication. The patient did undergo computed tomography angiography (CTA) of the extremities, which showed three-vessel runoff to the level of the malleolus with a transection of the dorsalis pedis. The orthopedic surgery team took the patient to the operating room where he underwent debridement of nonviable tissues with reduction of the fractures and placement of a negative pressure wound therapy system. Postoperatively the patient’s extremity was warm with palpable dorsalis pedis pulse. During rounds on hospital day three, the patient was noted to have a cool and pulseless foot distal to the malleolus, which is suggestive of ischemia. Therefore, the patient was taken urgently to the operating room by trauma surgery service. His preoperative wound is shown in Figure 2.

**Figure 3:**
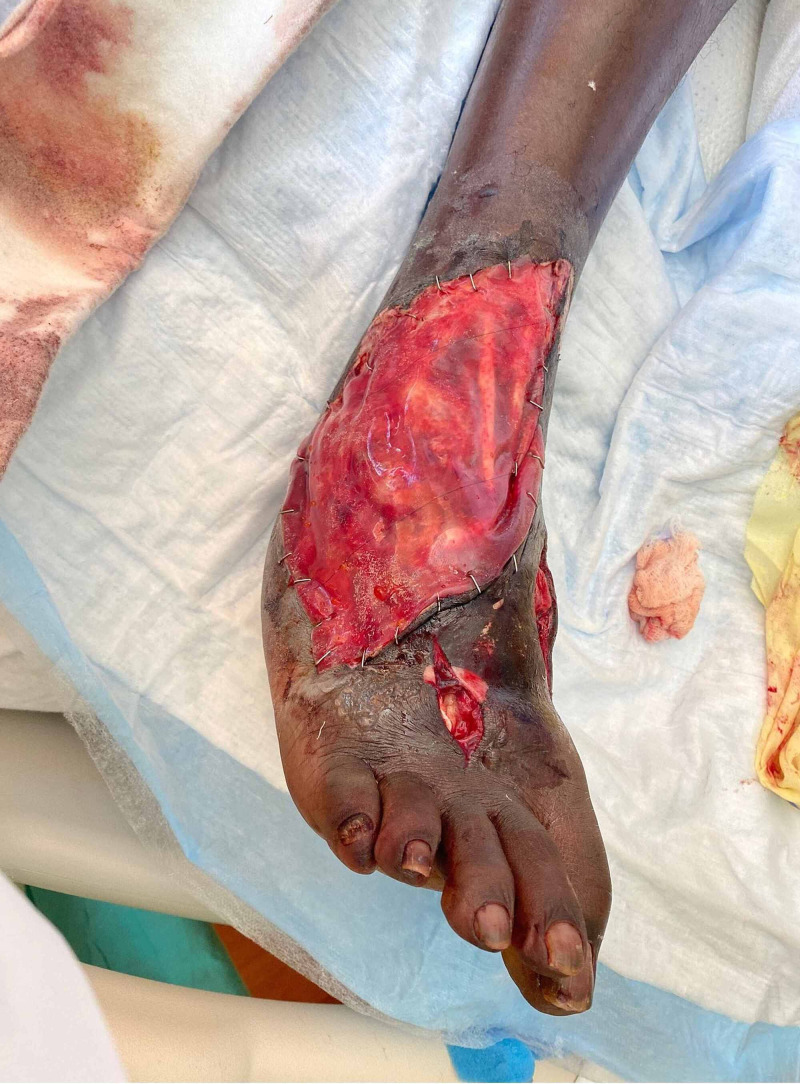
Patient's right lower extremity after revascularization and compartment release Shown here is the patient's right lower extremity after revascularization of the dorsalis pedis with a saphenous vein interposition graft and coverage with Integra^TM ^(Integra LifeSciences, Princeton, NJ). Compartment release was performed in the interdigit space. At this time the tissue was dusky but with Doppler signals in the first interdigit webspace.

In the operating room, the dorsalis pedis artery was exposed at the level of the ankle as identified by Doppler signals. This was exposed distally until a complete transection was identified 2 cm distal to the ankle. The distal portion was identified proximal to the webspace. Necrotic tissues were resected, and the vessel underwent embolectomy to restore brisk bleeding from the proximal segment before flushing and systemic heparinization. Distal greater saphenous vein was dissected, and an 8-cm segment was utilized to create a reversed interposition graft with spatulation of the ends. Fasciotomies were performed in the forefoot, and hematomas were evacuated from the compartments due to the presence of compartment syndrome. The wounds were packed with gauze, and, given the lack of nearby viable tissue for complete coverage, the exposed vessels and anastomosis were covered with Integra^TM^ (Integra LifeSciences, Princeton, NJ) before wrapping in bismuth-laden petroleum gauze and dry gauze. Postoperative images of the patient’s revascularized wound are shown in Figure 3.

**Figure 4:**
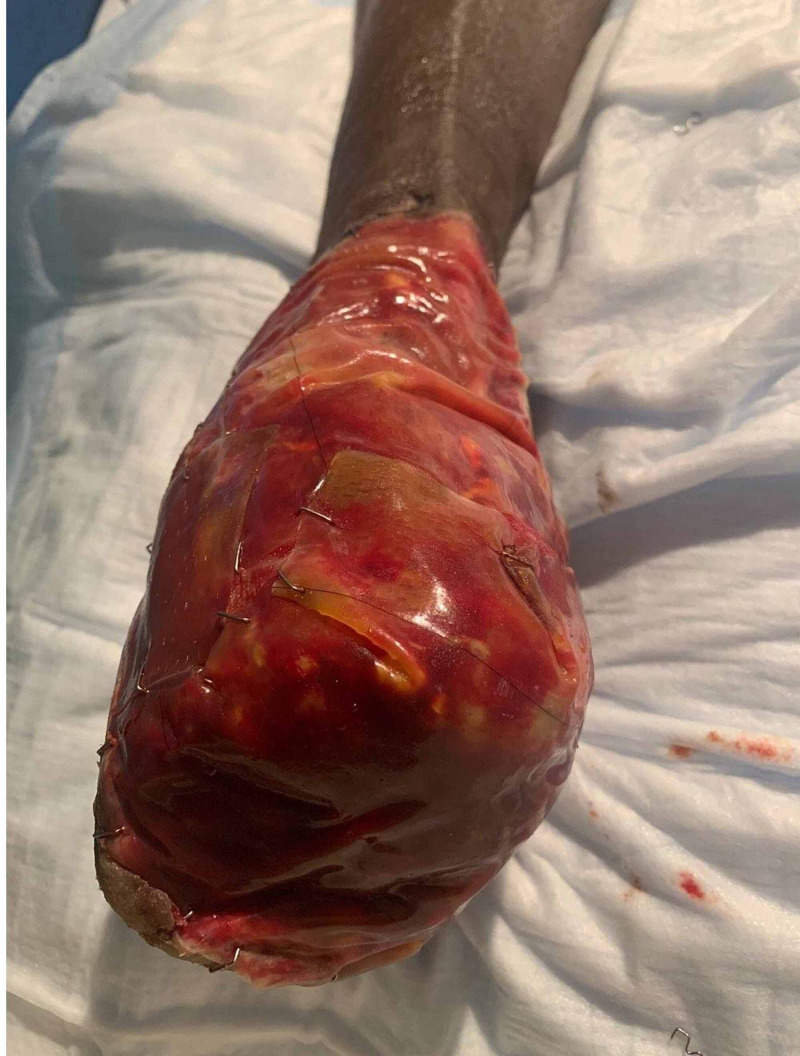
Patient's right lower extremity after transmetatarsal amputation Here is the remnant of the patient's right lower extremity after transmetatarsal amputation. Despite initial loss of all vascular supply distal to the malleolus the patient was able to have partial extremity salvage due. The wound here has maintained coverage with skin substitute due to lack of available viable tissue.

Postoperatively, the patient’s graft was maintained on a heparin infusion. His distal digits remained ischemic, but the patient had palpable pulses distally. Given the concern for continued ischemic insult with disruption of the microvasculature due to crush injury, the patient underwent hyperbaric therapy. Postoperative pictures of the wound are shown in Figure 4. Despite attempts to salvage the forefoot with continued hyperbaric therapy and serial debridement, the patient did require transmetatarsal amputation as shown in Figure 4 on the 19th hospital day before and after Integra^TM^ placement. The residual foot (and ankle) has remained viable and functional.

## Discussion

Primary dorsalis pedis reconstruction is not well described in the current literature. There are several case reports that describe aneurysmal disease after a traumatic crush injury, arterial access complications, and even attributed to repetitive footwear trauma [12-16]. Long-term patency conclusions from this data are difficult to infer; however, saphenous vein bypass to the dorsalis pedis in atherosclerotic disease has been described. In one of the largest retrospective studies of >1000 patients, the primary graft utilized was saphenous vein grafts (reversed and nonreversed) with primary patency rates of 56.8% and limb salvage rates of 78.2% [17]. It is unclear whether this long-term data is applicable to traumatic limb salvage.

In the authors’ review of the literature, the description of dorsalis pedis reconstruction in traumatic arterial transection has not been previously described, regardless of the mechanism of injury. Given the relative lack of anatomic variants within this region with reported dorsalis pedis arterial diameters of 1.9-3.4 mm, including the venous interposition graft for this type of distal extremity trauma into the surgeon’s armamentarium is noteworthy. Further, the description of compartment syndrome encountered intraoperatively within this patient is also unique. A recent review of current literature on the subject highlighted the heterogeneity in the reported number of compartments and their surgical decompression [18]. In this case study, based on our intraoperative finding of intramuscular hematomas and nonviable tissues within the foot we did create several incisions. While the patient did require transmetatarsal amputation, the level of limb salvage was improved through the attempted limb salvage techniques described here.

## Conclusions

Foot crush injury is a difficult problem both from the complexity of the injury pattern standpoint and also the significant clinical and socioeconomic burden that it represents to the patient. While scoring systems exist to predict limb salvage, the accuracy and implementation of these are varied, and thus clinical judgment must always be employed when attempting limb salvage. We describe the first reported use of a reversed saphenous interposition graft repair of a transected dorsalis pedis in a patient with complex open fractures of the distal tibia and metatarsals in an attempt at limb salvage. While the patient ultimately required transmetatarsal amputation, the authors of this report feel that this intervention may have prevented a below-the-knee amputation and potentially may have a more significant benefit if introduced early in the patient’s treatment.
